# Complete Genome Sequence of *Bacillus anthracis* HYU01, Isolated from Soil Samples in the Korean Peninsula

**DOI:** 10.1128/genomeA.00769-14

**Published:** 2014-08-07

**Authors:** Se Kye Kim, Won-Hyong Chung, Sang Hoon Kim, Kyoung Hwa Jung, Namshin Kim, Young Gyu Chai

**Affiliations:** a Department of Molecular and Life Science, Hanyang University, Ansan, Republic of Korea; b Korean Bioinformation Center, Korea Research Institute of Bioscience and Biotechnology, Daejeon, Republic of Korea; c Department of Nanobiotechnology, Hanyang University, Seoul, Republic of Korea

## Abstract

*Bacillus anthracis* is a Gram-positive endospore-forming bacterium that causes the zoonotic disease anthrax. We report a complete genome sequence of *B. anthracis* strain HYU01, isolated from Changnyung, which belongs to the B branch (B.Br.) 001/002 canonical single nucleotide polymorphism (canSNP) group.

## GENOME ANNOUNCEMENT

*Bacillus anthracis*, the causative agent of the zoonotic disease anthrax, is a low-G+C-content Gram-positive bacterium that has been a major concern in the field of livestock production and biodefense. *B. anthracis* is known for its high lethality and its resistance to various environmental factors. A tripartite toxin and a poly-glutamic acid capsule, encoded by two large plasmids (pXO1 and pXO2), are the principal virulence factors and are required for full virulence (1). Complete genome sequencing of *B. anthracis* Ames and other *Bacillus* groups was done, and the sequences provided useful information for the study of putative virulence factors and identification of therapeutic targets (2). Various genotyping techniques like canonical single nucleotide polymorphisms (canSNPs) have been developed to distinguish genetic relationship of *B. anthracis* according to their phylogenic properties. There are three major phylogenetic lineages (braches A, B, and C), further divided into 12 clonal sublineages by canSNP grouping (3). In an effort to further analyze our results from genetic populations of *B. anthracis* in Korea, we sequenced the genome of *B. anthracis* HYU01, previously reported as number 24, isolated from soil samples in Changnyung, Republic of Korea. Genotypic analyses showed in the previous study that HYU01 belonged to the B branch (B.Br.) 001/002 canSNP subgroup, which is rather uncommon in eastern Asia (4).

The whole genome of *B. anthracis* HYU01 was sequenced using a 454 GS-FLX (~93.8 Mb; 5-kb paired library) and an Illumina genome analyzer (~595 Mb; 200-bp paired library). Pretreatment of the sequenced reads and *de novo* assembly using the combination of SOAPdenovo 1.05 (5) and Newbler 2.6 (6) produced two scaffolds (38 contigs) and one contig. All gaps between contigs were closed by primer walking. The whole sequenced genome had 102-fold, 410-fold, and 180-fold coverage of the chromosome and pXO1 and pXO2, respectively. The coverage level in the genome sequence indicates a rough chromosome/pXO1/pXO2 molecular ratio of 1:4:2, implying that HYU01 has a greater coverage level than those of Ames Ancestor (1:3:2) for the pXO1 plasmid (7). The genome of HYU01 includes one circular chromosome (5,213,498 bp; G+C content, 35.4%), and two circular plasmids, pXO1 (181,894 bp; G+C content, 32.5%) and pXO2 (94,732 bp; G+C content, 33.1%). The genome sequence of HYU01 is 99.8% homologous to Ames Ancestor (chromosome, 99.8%; pXO1, 99.7%; and pXO2, 99.7%). HYU01 has a smaller chromosome size than Ames Ancestor, as short as ~2.9 kb. However, we found no large structural variations (>200-bp indel, inversion, and rearrangement) between HYU01 and Ames Ancestor.

Predictions of coding sequences (CDS) and annotations of their functions were performed with the RAST annotation system (8) and NCBI Prokaryotic Genome Annotation Pipeline (PGAP) (9). The chromosome of HYU01 contained 5,705 of the predicted genes (5,579 CDS and 126 RNAs) and two plasmids, pXO1 and pXO2, contained 185 and 103 CDS, respectively. Among the predicted 5,993 genes, 96 tRNA genes encompassing all 20 amino acids were identified using tRNAscan-SE (10). Ten rRNA operons were identified using RAST. All RNAs were identified in the chromosome.

The differences in the canSNP subgroups and the molecular ratios of pathogenicity plasmids may impose differences between *B. anthracis* HYU01 and Ames Ancestor.

### Nucleotide sequence accession numbers.

The whole-genome sequences of the *B. anthracis* HYU01, including those for pXO1 and pXO2, have been deposited in the GenBank database under accession numbers CP008846 (chromosome), CP008847 (pXO1), and CP008848 (pXO2).
